# The current role of telomerase in the diagnosis of bladder cancer

**DOI:** 10.4103/0970-1591.45535

**Published:** 2009

**Authors:** Sara Bravaccini, Valentina Casadio, Dino Amadori, Daniele Calistri, Rosella Silvestrini

**Affiliations:** Istituto Scientifico Romagnolo per lo Studio e la Cura dei Tumori (I.R.S.T.), Via Piero Maroncelli 40, 47014 Meldola (FC), Italy

**Keywords:** Bladder cancer, early diagnosis, telomerase, telomerase repeat amplification protocol

## Abstract

Bladder cancer has an incidence of 15 cases per 100,000 persons in the global population and is the most common tumor of the urinary tract. Imaging techniques, cytoscopy, and cytology are either invasive or not sufficiently accurate to detect early stage tumors, and the need for new diagnostic markers still remains. Among the markers most recently proposed to improve diagnostic accuracy and especially sensitivity, increasing attention has been focused on the role of the ribonucleoprotein, telomerase. Relevant papers on the etiology, diagnosis, and evaluation of bladder cancer using telomerase in urine were searched for and considered. The PubMed search was performed using the text terms “bladder cancer”, “diagnosis”, and “telomerase”. Previous studies have shown that the quantitative Telomerase Repeat Amplification Protocol (TRAP) assay performed in voided urine is an important non-invasive tool for the diagnosis of bladder tumors since it has very high sensitivity and specificity, even for early stage and low grade tumors. The main limitation of this test is the rate of false positive results due to the presence of inflammatory or non-tumor cells (i.e., epithelial cells from the lower genital tract), which express telomerase activity (TA). Consequently, an *in situ* analysis would seem to be important to identify the nature of telomerase-positive cells. Immunocytochemical detection of the hTERT subunit by a specific antibody seemed to open up the possibility to identify different cellular components of urine. However, the lack of a strict relationship between hTERT protein expression and telomerase activity has, to a certain extent, made this approach less relevant. In conclusion, telomerase activity in urine determined by TRAP seems to be marker of great potential, even more advantageous in cost/benefit terms when used in selected symptomatic patients or professionally high-risk subgroups.

## INTRODUCTION

Bladder cancer has an incidence of 15 cases per 100,000 persons in the global population with more than 60,000 new cases reported each year in the United States alone, and represents the fourth most common malignancy in men and the tenth in women.[1] It is the most commontumor of the urinary tract, after prostatic carcinoma, and it is between three- to seven-fold more frequent in males than in females.[2] About 90% of bladder malignancies are urothelial carcinomas, characterized by proliferation of the transitional epithelium (transitional cell carcinomas) and in about 25% of cases it is a multifocal disease.

Of particular aetiological importance is a history of exposure to chemical substances, which, as carcinogens or co-carcinogens, may lead to the development of carcinoma with a latency of up to 30 years. Recreational poisons, such as tobacco, have been implicated, and the role of industrial carcinogens has been recognized for a long time.[3] Besides chemical substances, other iatrogenic causes include medical radiation treatments of the lower pelvic region. Chronic cystitis has also been suspected, as well as schistosoma haematobium infections which are thought to be involved in squamous cell carcinoma. The cardinal, and first, symptom of bladder carcinoma is usually macrohaematuria. Indeed, any episode of painless macrohaematuria could suggest the presence of malignant urinary tract disease until proven otherwise. The staging of urothelial bladder carcinoma is based on the International Union Against Cancer (UICC) TNM classification, and on tumor cell differentiation (grading). Clinically relevant to prognosis at the time of diagnosis is whether the tumor is superficial, or has already invaded the underlying mucosa, as observed in about 30% of cases.

Bladder cancer is undoubtedly a tumor type that could benefit from screening as early detection has been demonstrated to greatly reduce mortality. In fact, survival is stage-dependent, and the five-year survival for patients with tumors confined to the mucosa is significantly higher than that of patients with muscle-invasive or metastatic cancers.[4]

Downstaging of bladder cancer through screening programs was first demonstrated more than ten years ago by Messing and co-workers[5] and was recently confirmed in the same case series in a 14-year follow-up.[6] In this study, the proportion of muscle-invasive tumors was significantly lower in screened (10%) than in unscreened males (60%). Moreover, whilst 20% of the unscreened population died from bladder cancer during follow-up, no deaths were observed in the group with screening-detected tumors.

The search for and development of an ideal marker for the early detection of bladder cancer has been intensely pursued in recent years, and a spectrum of markers has been identified and investigated. In particular, an ideal diagnostic test should be non-invasive, inexpensive, easy to perform, and the marker evaluated should be detectable in early stage and grade tumors such as *in situ* carcinoma. In addition, the test should be highly accurate to reduce the rate of false positive and negative results.

## DIAGNOSTIC TESTS CURRENTLY USED

Imaging techniques such as ultrasound (US), computed tomography (CT), and magnetic resonance imaging (MRI) are widely used for bladder cancer diagnosis. However, due to tumor size and localization they are not accurate enough to detect the majority of tumors, or to correctly distinguish between non-malignant lesions and reactive processes.[7] On the other hand, the invasive cystoscopy method is not able to detect tumors which remain below the mucosa surface, such as *in situ* carcinoma, nor to correctly interpret non-specific areas of redness.[7] However, notwithstanding these limitations, cystoscopy still represents the gold standard for bladder cancer detection with sensitivity and specificity rates ranging from 70 to 80%.[8] Bladder carcinoma recurrs in 70 to 80% of cases, and presents at a more advanced stage in 20 to 30%. A careful and frequent follow-up observation is therefore of paramount importance[9] and for this reason cystoscopic examinations are recommended every three months as standard practice.

More than 50 years ago, Papanicolaou and Marshall recognized the importance of a non-invasive technique for the diagnosis and follow-up of bladder carcinoma patients.[10] If such a method could also be cost-effective, its introduction as a screening method in at risk subgroups, including persons employed in textile, tannery, chemical, rubber, and pharmaceutical industries, as well as smokers, or in symptomatic individuals, could be useful.[7] However, non-invasive methods which are able to compete with cystoscopy in terms of diagnostic accuracy are still not available. For example, urine cytology examination is a simple test practicable in all laboratories, but despite its high specificity, it does not have sufficient sensitivity to accurately diagnose well-differentiated or early stage bladder carcinomas. In fact, while the specificity has been reported to vary from 84 to 100% in case-control studies, and from 93 to 99% in symptomatic patients, the sensitivity varies from 26 to 75% and from 16 to 56%, respectively [Table 1]. Moreover, cytologic examination is quite observer dependent, as shown by the high variability of interstudy results [Table 1] and for this reason it has become a less important diagnostic tool in recent years.

**Table 1:** Diagnostic accuracy of cytology

	Number of cases	Sensitivity (%)	Specificity (%)
**Case-control studies**			
Weikert *et al.*[38]	400	34	93
Halling *et al.*[12]	265	58	98
Babjuk *et al.*[22]	218	33	100
Eissa *et al.*[55]	200	75	94
Sarosdy *et al.*[11]	176	26	-
Eissa *et al.*[26]	168	44	100
May *et al.*[15]	166	71	84
Saad *et al.*[23]	120	48	87
Adb El Gawad *et al.*[21]	86	54	100
Placer *et al.*[13]	86	64	86
Varella-Garcia *et al.*[14]	19	43	100
Symptomatic patients			
Grossman *et al.*[27]	1331	16	99
Sarosdy *et al.*[20]	497	38	-
Laudadio *et al.*[19]	300	34	93
Sharma *et al.*[24]	278	56	93
Kavaler *et al.*[45]	151	51	98
Landman *et al.*[25]	77	40	94

Tabulated according to size of case-series

## NEW MOLECULAR NON-INVASIVE APPROACHES

The availability of more accurate diagnostic and possibly non-invasive tests has been a major objective pursued intensively in recent years. An ideal diagnostic marker should have both a high sensitivity and specificity, and also be able to detect well-differentiated and early stage tumors. The method must also be simple, and sufficiently inexpensive to facilitate the analysis of a large number of urine samples in a reasonable amount of time.

In recent years, several markers of diagnostic relevance have been identified and a number of reagents directed against molecular targets have been developed commercially [Table 2]. The most intensively investigated are chromosome alterations detected by fluorescence *in situ* hybridization (FISH),[11–20] urinary human complement factor H related protein (BTA stat and BTA TRAK),[11,18,21–25] nuclear matrix protein (NMP22),[21,23–28] followed by cytocheratin 8 and 18 fragments (UBC rapid, and UBC immunoradiometric assay, UBC ELISA).[15,22,26]

**Table 2:** Diagnostic accuracy of different non-invasive assays

	Number of cases	Type of assay	Sensitivity (%)	Specificity (%)
**Case-control studies**			
Halling *et al.*[12]	265	FISH	81	96
Skacel *et al.*[16]	120	FISH	85	97
Placer *et al.*[13]	86	FISH	80	85
Riesz *et al.*[17]	55	FISH	87	100
Varella-Garcia *et al.*[14]	19	FISH	87	100
Halling *et al.*[18]	265	FISH	81	100
		BTA stat	78	74
Sarosdy *et al.*[11]	176	FISH	71	100
		BTA stat	50	-
Saad *et al.*[23]	120	NMP22	81	87
		BTA stat	63	82
Babjuk *et al.*[22]	218	BTA stat	74	87
		BTA TRAK	76	73
		UBC rapid	49	79
		UBC IRMA	70	64
May *et al.*[15]	166	FISH	53	74
		UBC	40	75
Eissa *et al.*[26]	168	NMP22	85	91
		UBC	67	81
Adb El Gawad *et al.*[21]	86	NMP22	91	87
		BTA	100	92
Symptomatic patients				
Sarosdy *et al.*[20]	497	FISH	69	78
Laudadio *et al.*[19]	300	FISH	73	65
Grossman *et al.*[27]	1331	NMP22	56	86
Sharma *et al.*[24]	278	NMP22	82	82
		BTA stat	68	82
Atsü *et al.*[28]	82	NMP22	78	66
Landman *et al.*[25]	77	BTA	40	73
		NMP22	81	77

FISH = fluorescence *in situ* hybridization, BTA = bladder tumor antigen, NMP22 = nuclear matrix protein, UBC = urinary bladder cancer Tabulated according to size of case-series within each marker

With regard to the most intensively investigated markers, consistent results have been obtained for FISH, with a sensitivity of approximately 80%, and a specificity between 90 and 100% in case-control studies. However, the test is expensive, cannot be performed in all laboratories, and accuracy strongly decreases when it is used for symptomatic patients. FISH, like cytology, requires specialized personnel to ensure a correct morphologic evaluation. Similar sensitivity and specificity have been reported for NMP22 in case control studies, albeit with lower accuracy, especially in terms of sensitivity in symptomatic patients. For all these molecular tests, sensitivity ranges from 40 to 100% in different case-control studies, and from 40 to 82% in symptomatic patient series. Specificity also varies markedly, from 64 to 100% in the former, and from 65 to 86% in the latter subgroups [Table 2].

Moreover, intra-assay variability is often higher than inter-assay variability, indicating a potential lack of standardization of technical aspects and preanalytical phases. Indeed, specific protocols and standards often adopted by individual laboratories determine a wide range of results which are not easily comparable.

## TELOMERASE

Among the markers most recently proposed to improve diagnostic accuracy, especially in terms of sensitivity, increasing attention has been focused on the role of the ribonucleoprotein, telomerase. This enzyme consists of three subunits: an RNA component (hTR), which acts as a template for DNA replication,[29] a telomerase associated protein (TP1)[30] of as yet unknown function, and the telomerase reverse transcriptase (hTERT), which is responsible for catalytic activity.[31] Telomerase activity (TA) has been detected in almost all malignant cells and tissues, and only very occasionally in normal somatic cells.[32–34]

The telomeric repeat amplification protocol assay (TRAP), a polymerase chain reaction (PCR) based method for detection of TA, has been available since 1994.[32] The introduction of this method is an important milestone in telomerase research and has become the standard method for studying the diagnostic relevance of this enzyme [Table 3].[34–37] TA has also been determined qualitatively and quantitatively using modified TRAP assays, for example TRAP scintillation proximity assay, TRAP-ELISA, fluorescent TRAP assay, TRAP hybridization assay, and bioluminescence linked with TRAP. Other methods have focused on the detection of the telomerase subunits, hTR and hTERT, using the reverse transcriptase polymerase chain reaction (RT-PCR). Real-time PCR methods have also permitted a quantitative and reproducible determination of these subunits.[38] Expression of the hTERT protein has also been analyzed by immunocytochemistry using anti-hTERT monoclonal[39,40] and polyclonal antibodies.[41]

**Table 3:** Diagnostic accuracy of telomerase-based assays

	Number of cases	Type of marker	Sensitivity (%)	Specificity (%)
Case-control studies				
Halling *et al.*[18]	265	TA*	46	91
Sanchini *et al.*[37]	218	TA*	90	88
Bravaccini *et al.*[47]	212	TA*	87	66
Sanchini *et al.*[36]	200	TA*	92	81
Saad *et al.*[23]	120	TA*	84	93
Fedriga *et al.*[35]	106	TA*	89	68
Adb El Gawad *et al.*[21]	86	TA*	80	95
Eissa *et al.*[55]	200	TA*	75	92
		hTERT	96	96
		HTR	92	89
Weikert *et al.*[38]	400	hTR	77	72
		hTERT	55	85
Symptomatic patients				
Kavaler *et al.*[45]	151	TA*	85	66
Landman *et al.*[25]	77	TA*	80	80

* TA performed by TRAP assay

## ENZYMATIC ACTIVITY

### TRAP assay

The detection of TA in bladder washing and voided urine has been investigated for its diagnostic potential. Since this technique detects TA, and not only the presence of the enzyme, viable cells are a prerequisite. In fact, a possible limitation of the TRAP assay is the potential vulnerability and inactivation of the enzyme by external factors.[7] Bladder washings are obtained by mechanical irrigation of the empty urinary bladder using saline solution at physiological pH. However, in native urine, suspended tumor cells are exposed to destructive substances such as proteases, urea, salts and, usually, acid pH, for variable times. All of these factors may lead to early inactivation or degradation of the enzyme that could explain the lack of reproducibility of results among the different studies. Moreover, bladder washings are obtained through the use of a catheter or cystoscope, which are both invasive instruments. For this reason, voided urine has been the most widely used biological sample for the TRAP assay.

The first reported TRAP assay studies were based on qualitative, and thereafter with semi-quantitative TA determinations.[42] To obtain more accurate and reliable results, a quantitative TRAP assay was developed in bladder washings and voided urine, based on exponential amplification of the primer-telomeric repeats generated in the telomerase reaction.[36,43–46] Several case-control studies have also confirmed that this test is more accurate in males than females,[36] with a higher specificity in younger than older individuals.[37] A recent study by the same authors suggested that these results could be due to the presence of inflammatory cells, which are almost always positive to telomerase.[47] Furthermore, the diagnostic accuracy of TA was not related to the tumor stage or grade, and was as high in both early stage and low grade tumors, including *in situ* carcinomas,[36] in contrast to what has been reported by other authors.[23] However, before introducing this test in routine clinical practice, in combination with, or as an alternative to invasive cytoscopy, its potential, in terms of sensitivity and specificity, must be further investigated and defined in a consecutive series of symptomatic individuals.[48]

## EXPRESSION OF HTR AND HTERT

### RT-PCR

It has been shown that transcriptional regulation of the catalytic component of the telomerase complex is a major determinant in the control of TA.[49,50] Meanwhile, hTR seems to be ubiquitously expressed in most cells,[29,51] independent of enzyme activity. Studies have pointed out that high hTERT mRNA expression is associated with malignancy in many tumor histotypes, and has shown great potential for early cancer detection in body fluids.[7,46,52] Indeed, the expression of hTERT and hTR mRNA, both in tissues[53] and in voided urine samples,[38] seems to correlate positively with tumor stage and grade, even if these data have not, as yet, been confirmed.[54] Moreover, a good concordance has been shown between mRNA of both telomerase subunits and telomerase activity.[55]

### Immunocytochemistry

**Figure 1:**
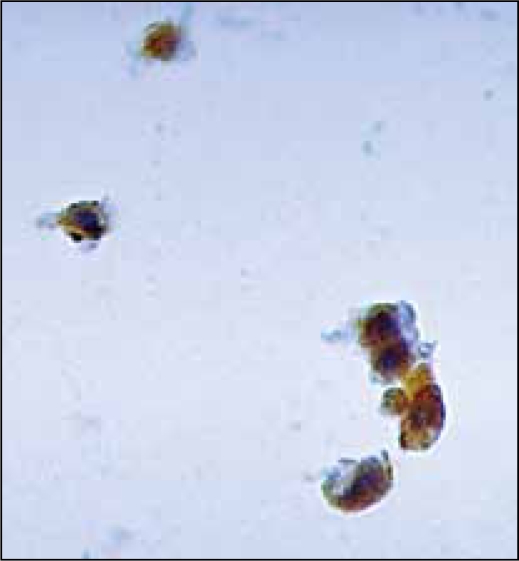
Immunoreactivity of bladder tumor cells to Mab anti-hTERT tel 3 36-10 Diesse

**Figure 2:**
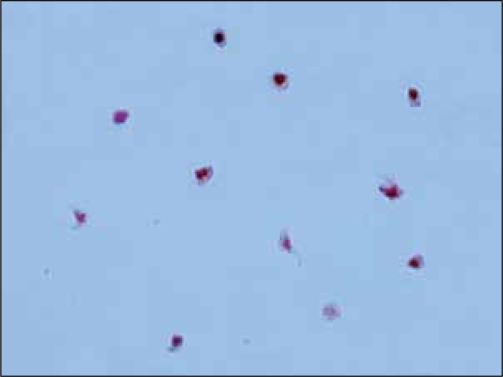
Immunoreactivity of inflammatory cells to Mab anti-hTERT tel 3 36-10 Diesse

Many studies have shown that the TRAP assay does indeed have some drawbacks, the most important being the rate of false positives due to the presence of inflammatory non-tumor cells in voided urine and bladder washings.[36,47] It is therefore important to carry out a morphological analysis to identify the true nature of urothelial telomerase expressing cells [Figure 1] and to unmask any false TRAP positives [Figure 2]. The availability of both monoclonal (Mab tel 3 36-10 DIESSE Diagnostica Senese Italy, commercialized by the Alexis Corporation, Lausanne, Switzerland; NCL-hTERT Novocastra, Newcastle- upon Tyne UK) [Figure 1] and polyclonal antibodies (TERT H-231: sc-7212, Santa Cruz Biotechnology Inc, Santa Cruz, CA, USA; hTERT EST21A Alpha Diagnostic International, San Antonio, TX), able to detect hTERT protein expression, has opened up the possibility of studying the different cell components. Up to now very few diagnostic studies on urine have been conducted using anti-hTERT antibodies; some have used freshly-filtered cytological samples,[36] while others have utilized sections of urine cells from paraffin-embedded blocks.[56] Depending on the antibody used, nuclear, nucleolar or cytoplasmic staining singly or in combination, were detected. Both nuclear and cytoplasmic hTERT positivity has been observed previously.[40] Indeed, the authors hypothesized that the positivity in the cytoplasm could be due to either a disruption of the normal hTERT nuclear translocation process during malignant transformation, or to the existence of post-transcriptional/post-translational modes of telomerase regulation such as hTERT phosphorylation, which are responsible for telomerase structure and activity. Furthermore, since the enzyme forms a large dimer/multidimer complex, correct assembly of the different components is important for catalytic activity. Almost all published studies have consistently shown the frequent or almost total presence of positivity in inflammatory cells [Figure 2]. In addition, an accurate analysis of anti hTERT antibody (tel 3 36-10) determinations[36] has shown a higher fraction of immunoreactive inflammatory and non bladder epithelial cells in women than in men. This finding has been suggested to be due to the shorter female urethra, which favors the entrance of bacteria into the bladder and could, at least in part, explain the increased number of false positive results.[47] The diagnostic accuracy of the TRAP assay could be improved by considering the percentage of non-tumor hTERT-expressing cells in the same urine sample. However, it still needs to be demonstrated that the two markers are equivalent. In fact, there is evidence that some tissues may be positive for hTERT mRNA, but not for TA.[57]

## CONCLUSIONS

The importance of early diagnosis for bladder cancer has undoubtedly been demonstrated. Most of the diagnostic approaches currently used are either invasive or do not assure sufficient accuracy, especially in terms of sensitivity. Among the non-invasive approaches, urine cytology presents major limitations in detecting tumors of low stage and grade. An ideal test should be non-invasive, accurate, easy to perform and reproducible. Moreover, due to the relatively low incidence of bladder tumors in the general population, the test should be used to screen professionally high-risk groups, or symptomatic patients, 5 to 10% of which present with bladder cancer, to be advantageous in cost/benefit terms.[48] The urine telomerase assay satisfies many of these requirements, providing a good sensitivity and specificity in case-control studies and a somewhat lower but acceptable sensitivity and specificity in the few studies performed on symptomatic patients. The main limitation of this marker is the presence of false positives due to the telomerase activity (TA) inherent within inflammatory and/or non-urothelial cells in urine. Consequently, an *in situ* analysis would appear essential to reduce the number of false TRAP positive results.[36,47] Evaluation of the intrinsic RNA component (hTR) by *in situ* hybridization, as well as immunocytochemical assessment of hTERT subunit expression, have both been applied as surrogate markers of TA. However, hTR evaluation by *in situ* hybridization makes quantitative analysis difficult and not all pathology laboratories are suitably equipped to perform this method. In contrast, the availability of anti-hTERT antibodies has opened up the possibility to easily identify the different cellular components of urine. Nonetheless, there is some doubt on the feasibility of immunocytochemical hTERT protein detection since the presence of the protein is not necessarily associated with its activity.[40]

Urine TA appears to show great potential as an early diagnostic marker, particularly if used in high-risk professional groups[48] and symptomatic patients. In any case, further prospective studies are needed to fully demonstrate its suitability as a first-line diagnostic tool. Other specific markers should also be investigated, for example, chromosomal alterations by fluorescence *in situ* hybridization that have maximum specificity, and could therefore be a second level diagnostic approach for unmasking false positive TRAP results and increasing the diagnostic accuracy.

## References

[1] American Cancer Society (2004). *Cancer Facts and Figures 2004*.

[2] Parkin DM, Bray F, Ferlay J, Pisani P (2005). Global cancer statistics, 2002. CA Cancer J Clin.

[3] Rehn L (1895). Blasengeschwülste bei Fuchsin-arbeitern. Arch Clin Chir.

[4] American College of Surgeons Commission on Cancer National Cancer Database.

[5] Messing EM, Young TB, Hunt VB, Gilchrist KW, Newton MA, Bram LL (1995). Comparison of bladder cancer outcome in men undergoing hematuria home screening versus those with standard clinical presentations. Urology.

[6] Messing EM, Madeb R, Young T, Gilchrist KW, Bram L, Greenberg EB (2006). Long-term outcome of hematuria home screening for bladder cancer in men. Cancer.

[7] Müller M (2002). Telomerase: Its clinical relevance in the diagnosis of bladder cancer. Oncogene.

[8] Kriegmair M, Baumgartner R, Knüchel R, Stepp H, Hofstädter F, Hofstetter A (1996). Detection of early bladder cancer by 5-aminolevulinic acid induced porphyrin fluorescence J Urol.

[9] Abel PD (1988). Prognostic indices in transitional cell carcinoma of the bladder. Br J Urol.

[10] Papanicolaou GN, Marshall VF (1945). Urine sediment smears as a diagnostic procedure in cancers of the urinary tract. Science.

[11] Sarosdy MF, Schellhammer P, Bokinsky G, Kahn P, Chao R, Yore L (2002). Clinical evaluation of a multi-target fluorescent *in situ* hybridization assay for detection of bladder cancer. J Urol.

[12] Halling KC, King W, Sokolova IA, Meyer RG, Burkhardt HM, Halling AC (2000). A comparison of cytology and fluorescence *in situ* hybridization for the detection of urothelial carcinoma. J Urol.

[13] Placer J, Espinet B, Salido M, Solé F, Gelabert-Mas A (2002). Clinical utility of a multiprobe FISH assay in voided urine specimens for the detection of bladder cancer and its recurrences, compared with urinary cytology. Eur Urol.

[14] Varella-Garcia M, Akduman B, Sunpaweravong P, Di Maria MV, Crawford ED (2004). The UroVysion fluorescence *in situ* hybridization assay is an effective tool for monitoring recurrence of bladder cancer. Urol Oncol.

[15] May M, Hakenberg OW, Gunia S, Pohling P, Helke C, Lübbe L (2007). Comparative diagnostic value of urine cytology, UBC-ELISA, and fluorescence *in situ* hybridization for detection of transitional cell carcinoma of urinary bladder in routine clinical practice. Urology.

[16] Skacel M, Fahmy M, Brainard JA, Pettay JD, Biscotti CV, Liou LS (2003). Multitarget fluorescence *in situ* hybridization assay detects transitional cell carcinoma in the majority of patients with bladder cancer and atypical or negative urine cytology. J Urol.

[17] Riesz P, Lotz G, Páska C, Szendrôi A, Majoros A, Németh Z (2007). Detection of Bladder Cancer from the urine using fluorescence *in situ* hybridization technique. Pathol Oncol Res.

[18] Halling KC, King W, Sokolova IA, Karnes RJ, Meyer RG, Powell EL (2002). A comparison of BTA stat, hemoglobin dipstick, telomerase and Vysis Urovysion assays for the detection of urothelial carcinoma in urine. J Urol.

[19] Laudadio J, Keane TE, Reeves HM, Savage SJ, Hoda RS, Lage JM (2005). Fluorescence *in situ* hybridization for detecting transitional cell carcinoma: Implications for clinical practice. BJU Int.

[20] Sarosdy MF, Kahn PR, Ziffer MD, Love WR, Barkin J, Abara EO (2006). Use of a multitarget fluorescence *in situ* hybridization assay to diagnose bladder cancer in patients with hematuria. J Urol.

[21] Adb El Gawad IA, Moussa HS, Nasr MI, El Gemae EH, Masooud AM, Ibrahim IK (2005). Comparative study of NMP-22, telomerase and BTA in the detection of bladder cancer. J Egypt Natl Canc Inst.

[22] Babjuk M, Kostíroví M, Mudra K, Pecher S, Smolová H, Pecen L (2002). Qualitative and quantitative detection of urinary human complement factor H-related protein (BTA stat and BTA TRAK) and fragments of cytokeratins 8, 18 (UBC rapid and UBC IRMA) as markers for transitional cell carcinoma of the bladder. Eur Urol.

[23] Saad A, Hanbury DC, McNicholas TA, Boustead GB, Morgan S, Woodman AC (2002). A study comparing various noninvasive methods of detecting bladder cancer in urine. BJU Int.

[24] Sharma S, Zippe CD, Pandrangi L, Nelson D, Agarwal (1999). A Exclusion criteria enhance the specificity and positive predictive value of NMP22 and BTA stat. J Urol.

[25] Landman J, Chang Y, Kavaler E, Droller MJ (1998). Liu BC Sensitivity and specificity of NMP-22, telomerase, and BTA in the detection of human bladder cancer. Urology.

[26] Eissa S, Swellam M, Sadek M, Mourad MS, El Ahmady O, Khalifa (2002). A Comparative evaluation of the nuclear matrix protein, fibronectin, urinary bladder cancer antigen and voided urine cytology in the detection of bladder tumors. J Urol.

[27] Grossman HB, Messing E, Soloway M, Tomera K, Katz G, Berger Y (2005). Detection of bladder cancer using a point-of-care proteomic assay. JAMA.

[28] Atsü N, Ekici S, Oge O, Ergen A, Hasçelik G, Ozen H (2002). False-positive results of the NMP22 test due to hematuria. J Urol.

[29] Feng J, Funk WD, Wang SS, Weinrich SL, Avilion AA, Chiu CP (1995). The RNA component of human telomerase. Science.

[30] Harrington L, McPhail T, Mar V, Zhou W, Oulton R, Bass MB (1997). A mammalian telomerase-associated protein. Science.

[31] Nakamura TM, Morin GB, Chapman KB, Weinrich SL, Andrews WH (1997). Lingner J Telomerase catalytic subunit homologs from fission yeast and human. Science.

[32] Kim NW, Piatyszek MA, Prowse KR, Harley CB, West MD, Ho PL (1994). Specific association of human telomerase activity with immortal cells and cancer. Science.

[33] Wright WE, Piatyszek MA, Rainey WE, Byrd W, Shay JW (1996). Telomerase activity in human germline and embryonic tissues and cells. Dev Genet.

[34] Shay JW, Bacchetti S (1997). A survey of telomerase activity in human cancer. Eur J Cancer.

[35] Fedriga R, Gunelli R, Nanni O, Bacci F, Amadori D, Calistri D (2001). Telomerase activity detected by quantitative assay in bladder carcinoma and exfoliated cells in urine. Neoplasia.

[36] Sanchini MA, Bravaccini S, Medri L, Gunelli R, Nanni O, Monti F (2004). Urine telomerase: An important marker in the diagnosis of bladder cancer. Neoplasia.

[37] Sanchini MA, Gunelli R, Nanni O, Bravaccini S, Fabbri C, Sermasi A (2005). Relevance of urine telomerase in the diagnosis of bladder cancer. JAMA.

[38] Weikert S, Krause H, Wolff I, Christoph F, Schrader M, Emrich T (2005). Quantitative evaluation of telomerase subunits in urine as biomarkers for noninvasive detection of bladder cancer. Int J Cancer.

[39] Soldateschi D, Bravaccini S, Berti B, Brogi A, Benicchi T, Soldatini C (2005). Development and characterization of a monoclonal antibody directed against human telomerase reverse transcriptase (hTERT). J Biotechnol.

[40] Volpi A, Bravaccini S, Medri L, Cerasoli S, Gaudio M, Amadori D (2005). Usefulness of immunological detection of the human telomerase reverse transcriptase. Cell Oncol.

[41] Hiyama E, Hiyama K, Yokoyama T, Shay JW (2001). Immunohistochemical detection of telomerase (hTERT) protein in human cancer tissues and a subset of cells in normal tissues. Neoplasia.

[42] Yokota K, Kanda K, Inoue Y, Kanayama H, Kagawa S (1998). Semi-quantitative analysis of telomerase activity in exfoliated human urothelial cells and bladder transitional cell carcinoma. Br J Urol.

[43] Wright WE, Shay JW, Piatyszek MA (1995). Modifications of a telomeric repeat amplification protocol (TRAP) result in increased reliability, linearity and sensitivity. Nucleic Acids Res.

[44] Kim NW, Wu F (1997). Advances in quantification and characterization of telomerase activity by the telomeric repeat amplification protocol (TRAP). Nucleic Acids Res.

[45] Kavaler E, Landman J, Chang Y, Droller MJ, Liu BC (1998). Detecting human bladder carcinoma cells in voided urine samples by assaying for the presence of telomerase activity. Cancer.

[46] Gelmini S, Crisci A, Salvadori B, Pazzagli M, Selli C, Orlando C (2000). Comparison of telomerase activity in bladder carcinoma and exfoliated cells collected in urine and bladder washings, using a quantitative assay. Clin Cancer Res.

[47] Bravaccini S, Sanchini MA, Granato AM, Gunelli R, Nanni O, Amadori D (2007). Urine telomerase activity for the detection of bladder cancer in females. J Urol.

[48] Lotan Y, Svatek RS, Sagalowsky Al (2006). Should we screen for bladder cancer in a high-risk population? A cost per life-year saved analysis. Cancer.

[49] Cong YS, Wright WE, Shay JW (2002). Human telomerase and its regulation. Microbiol Mol Biol Rev.

[50] Horikawa I, Barrett JC (2003). Transcriptional regulation of the telomerase hTERT gene as a target for cellular and viral oncogenic mechanisms. Carcinogenesis.

[51] Meyerson M, Counter CM, Eaton EN, Ellisen LW, Steiner P, Caddle SD (1997). hEST2, the putative human telomerase catalytic subunit gene, is up-regulated in tumor cells and during immortalization. Cell.

[52] de Kok JB, Ruers TJ, van Muijen GN, van Bokhoven A, Willems HL, Swinkels DW (2000). Real-time quantification of human telomerase reverse transcriptase mRNA in tumors and healthy tissues. Clin Chem.

[53] Takihana Y, Tsuchida T, Fukasawa M, Araki I, Tanabe N, Takeda M (2006). Real-time quantitative analysis for human telomerase reverse transcriptase mRNA and human telomerase RNA component mRNA expressions as markers for clinicopathologic parameters in urinary bladder cancer. Int J Urol.

[54] Bowles L, Bialkowska-Hobrzanska H, Bukala B, Nott L, Razvi H (2004). A prospective evaluation of the diagnostic and potential prognostic utility of urinary human telomerase reverse transcriptase mRNA in patients with bladder cancer. Can J Urol.

[55] Eissa S, Swellam M, Ali-Labib R, Mansour A, El-Malt O, Tash FM (2007). Detection of telomerase in urine by 3 methods: Evaluation of diagnostic accuracy for bladder cancer. J Urol.

[56] Khalbuss W, Goodison S (2006). Immunohistochemical detection of hTERT in urothelial lesions: A potential adjunct to urine cytology. Cytojournal.

[57] Liu K, Hodes RJ, Weng Np (2001). Cutting edge: Telomerase activation in human T lymphocytes does not require increase in telomerase reverse transcriptase (hTERT) protein but is associated with hTERT phosphorylation and nuclear translocation. Immunol.

